# Championing reproductive and perinatal health with the recovery community: improving access to healthcare and health promotion resources to support recovery

**DOI:** 10.3389/fpubh.2025.1529169

**Published:** 2025-05-14

**Authors:** Hartley Feld, Alex Elswick, Jeremy Byard, Whitney Beckett, Amanda Fallin-Bennett

**Affiliations:** ^1^College of Nursing, University of Kentucky, Lexington, KY, United States; ^2^College of Agriculture, Food and Environment, University of Kentucky, Lexington, KY, United States

**Keywords:** recovery, reproductive health, pregnancy, substance use disorder, community-engaged, pregnancy intention

## Abstract

Peer recovery support services are instrumental in the promotion of long-term recovery primarily by focusing on building the recovery capital of people with substance use disorders. Women may have specific health-related needs that are not generally part of recovery support staff training. Our team co-created a model by training people with lived experience as coaches to promote the health of women with SUD during the critical period of their reproductive years when mortality from overdose risk is high and can be compounded by issues surrounding pregnancy. We explored the outcomes of a small pilot test of this model to promote reproductive autonomy in a recovery community center (RCC). The RCC and the champion-trained peer recovery coach were able to increase their reach to women of reproductive age and facilitated linkage to healthcare and health-promoting resources. The model has the potential to improve the participants' abilities to access reproductive and perinatal health resources and healthcare that could lead to improvements in their recovery.

## Introduction

Recovery capital (RC) is a biopsychosocial framework effectively applied for recovery from substance use disorder (SUD). Developed by Granfield and Cloud (1), RC is an asset-based framework that refers to the resources, internal and external to an individual, that can be mobilized to support recovery (2). RC is most often conceptualized as an ecological model that addresses individual (e.g., transportation, employment, housing), social (e.g., professional support, family and friends), and community/cultural-level factors (e.g., community attitudes toward SUD, treatment accessibility) (3). Research has revealed that RC is associated with sustained recovery, higher quality of life, and reduced biopsychosocial stress (4). In a recent update on the science of RC, Best and Hennesy posited that RC “meet(s) an individual where they are at within their larger contextual environment…and acknowledges the social determinants of health and how they may influence substance use” [(5), p. 3]. Therefore, RC is uniquely suited to the study of social intersections [i.e., gender, race, socioeconomic status (SES), etc.] and substance use.

Although the literature on RC is still evolving, some prior research has examined the relationship between RC and gender (6–8). Women face unique barriers to SUD recovery that result in the accumulation of greater “negative recovery capital”, as compared to men (9). Specifically, women are more likely to report experiencing greater levels of stigma and judgment, and gender-specific barriers to RC such as childcare and other unmet service needs than men (10, 11). In their recent analysis of differences in RC accumulation as stratified by gender, Abreu-Minero et al. (6) found that despite reporting higher levels of general health management, women were more likely to have residual mental health problems and experience a higher level of domestic violence than men. Moreover, the most significant predictor of RC growth among women in that study was general health management, which suggests that health may be more closely related to overall RC for women as compared to men. There are also gaps in the literature as the complex health-related RC needs of pregnancy-capable people who do not identify as women, such as trans men and non-binary people, have not been paid enough attention (12). However, for the purpose of this study, our focus is on examining the binary historical literature as it relates to RC issues of women with SUD and women of reproductive age who have the potential for pregnancy.

Reproductive and perinatal health are critically underexplored components of women's RC, and public health challenges surrounding pregnancy and SUD are immense. During maternal care hospitalization, women with an opioid use disorder have a 4.6-fold increased risk of maternal death, contributing to the high rates of maternal mortality in the United States (13, 14). Although there have been numerous efforts to improve maternal healthcare surrounding delivery and birth, over 60% of all maternal deaths occur outside of healthcare settings within a year of delivery and are largely related to non-obstetric causes and social determinants of health (15, 16). These socially or behaviorally driven deaths are categorized as pregnancy-associated deaths, such as those due to overdose, suicide, and homicide. Pregnancy-associated maternal deaths due to overdose rose by 81% between 2017 and 2020, significantly contributing to disparate maternal mortality rates in the United States and pointing to the urgent need for interventions to improve perinatal and reproductive healthcare access for people with SUD (17).

Women who use drugs face myriad barriers to accessing reproductive and perinatal healthcare, which causes disparities in morbidity and mortality, such as fear of the involvement of child protective services or stigma, limited transportation, and a lack of knowledge of service options (18, 19). These challenges can be further compounded in case of an unintended pregnancy. Women who use drugs report higher rates of unintended pregnancy (>75%) and initiate prenatal care later than women who do not use drugs (6, 20, 21). People in SUD treatment report unplanned or unintended pregnancies due to lack of reproductive health knowledge, low levels of contraceptive use, low levels of social support, and limited access to reproductive health services (22–24). Having an unintended pregnancy exacerbates the existing mental health challenges and erodes recovery efforts by increasing stress, anxiety, and depression (25–27). Also, unintended pregnancy is associated with partner violence, and people with SUD are three to five times more likely to be victimized by violence than those without SUD (28, 29). Additionally, women with SUD are more likely to have rapid repeat pregnancies (< 18 months between) which is associated with adverse maternal/child health outcomes (30). Furthermore, the policy environment limiting or eliminating abortion care in states such as Kentucky (where the current pilot takes place) made the impetus for preventing unwanted pregnancy even more imperative and timely. Limited data exists about the prevalence of abortion of people with an SUD; in one study prior to the Dobbs decision, 42% of people seeking harm reduction services report having at least one abortion (31).

The alarming rates of maternal overdose, mortality, unintended pregnancy, and rapid repeat pregnancy reflect the limited access to and engagement with healthcare (32). Furthermore, state policies designed to reduce perinatal substance use (such as classifying illicit drug use during pregnancy as child abuse) have not been effective and undermine healthcare engagement, leading to significantly later initiation of prenatal care and fewer women receiving post-partum care as compared to states without these policies (33–35). Seeking and engaging in healthcare has a protective effect and confers even greater benefits to women with chronic diseases, such as SUD (36, 37). Preconception and perinatal healthcare reduce not only maternal mortality but also birth defects, pre-term birth, and infant mortality and has the greatest benefit for women with chronic diseases and marginalized populations (such as people with ethnic minoritized identities, those who are stigmatized due to substance use, or are from under-resourced communities) (38).

## Background

### Reproductive autonomy

Reproductive autonomy is defined as the power to control and decide when and if to use contraception, become pregnant, and bear children (39, 40). It can be undermined by limiting reproductive rights, coercion (by partner, provider, researcher), disempowerment, and poor communication with sexual partners (41–43). Historically, marginalized women with SUDs have been unjustly targeted for forced or coerced sterilization as a part of widespread eugenics movements. They were targeted as a means to reduce the growth of families whose mothers were deemed “feebleminded, defective, and criminal” (44). More recently, potentially coercive tactics targeting women with SUD included offering cash incentives for the most effective forms of contraception, contributing to the perception that preventing pregnancy is the primary goal (39, 40). Public health researchers have also largely focused on increasing the initiation of the most effective methods of contraception, such as long-acting contraception (45). However, due to historical injustices, marginalization, fear, and stigma, a more nuanced approach is needed to improve reproductive autonomy and access to a full range of contraceptive or family planning healthcare options. Our previous qualitative study revealed that some people in recovery as well as some people who have issues of chaotic or problematic use who aren't ready to make longer term fertility decisions would appreciate low-barrier access to emergency contraception, as cost and stigma were identified as barriers (46, 47). Moreover, they may benefit from having emergency contraception before they need it. The advance provision of emergency contraception increases the odds that a woman will use it (2.5-fold) and speeds up the time from intercourse to use by 14.6 h as compared to control groups (48).

Little is known about how to effectively engage women with SUD for the promotion of reproductive autonomy, especially among women who want to become pregnant. Improving access to and engagement in preconception and early prenatal care have been identified as maternal/child health priorities (23, 49). Recent literature has emphasized the need for greater engagement in preconception health services and a more proactive approach to address SUD-related health issues before pregnancy (50). Women with SUD who would like to become pregnant could also benefit from an early awareness of a pregnancy, as a positive pregnancy test is associated with behavioral change and reduction in alcohol use, which can be protective against miscarriage in the first trimester and fetal alcohol spectrum disorders (45, 51). Additionally, consuming prenatal vitamins in the preconception period and in early pregnancy confers a protective effect against numerous types of birth defects (52). Pregnancy testing and obtaining prenatal vitamins are part of preconception and early prenatal healthcare, yet women with SUD are more than twice as likely to have only late prenatal care (third trimester only) or no prenatal care (40%) as compared to those without substance use during pregnancy (16%) (53). New community-driven approaches to engage women with SUD who would like to become pregnant or prevent pregnancy are critically needed to promote reproductive autonomy and empowerment to reduce adverse reproductive and maternal/child health outcomes (23, 49).

### Recovery community centers and peer-based recovery support services

Peer-based recovery support services are an emerging modality of availing recovery resources delivered in the community or clinical settings by trusted peers (people with lived experience) in formalized and specialized roles (54–56). One such peer-based recovery support service that is rapidly growing is recovery coaching within the Recovery Community Centers (RCC). Recovery coaches (also referred to as “peer support specialists” or “peer mentors”) are peers with lived experience in recovery who help people with SUDs build their recovery capital. Recovery coaches effectively build rapport and collaborative, supportive relationships with people with SUD to facilitate the latter's access to the needed resources (57). Researchers have observed that recovery coaching is associated with reduced substance use (58, 59) reduced odds of drinking to intoxication (60), improved stress management (61), decreased hospitalizations (62), decreased criminal justice involvement (59), and increased treatment adherence (63).

RCCs employ peer recovery coaches and are ideally positioned to offer programs that support reproductive or perinatal health. They are brick-and-mortar facilities serving as a community hub for recovery support services (64). RCCs typically provide a variety of services including drop-in social support, harm reduction services, and facilitated access to recovery capital via recovery coaching (65). These centers are intended for more continuous, ongoing engagement with people with SUD and people who use drugs (PWUDs). They are well positioned to fill important gaps because they are free, highly visible, and inclusive of all pathways of recovery, including harm reduction. While some PWUD may not have felt welcome in recovery communities where abstinence-only was the focus, RCCs prioritize co-location of harm reduction and recovery services to engage individuals across the spectrum of substance use and irrespective of stage of change (66).

RCCs have the potential to fill an important gap in the linkage to reproductive and perinatal healthcare, because current interventions are largely designed for people who are in treatment settings (20), leaving behind 91% of reproductive-age women who need treatment for SUD and who did not receive treatment in the last year (67). Furthermore, there is a need to develop recovery coaching programs designed to promote access to reproductive and perinatal health and healthcare. Though there are specialized programs in recovery, harm reduction, or peer coaching spaces that serve pregnant, parenting, and post-partum people, they are largely understudied and are generally not focused on reproductive health and wellbeing (68–70).

### Access to healthcare

**Figure 1:**
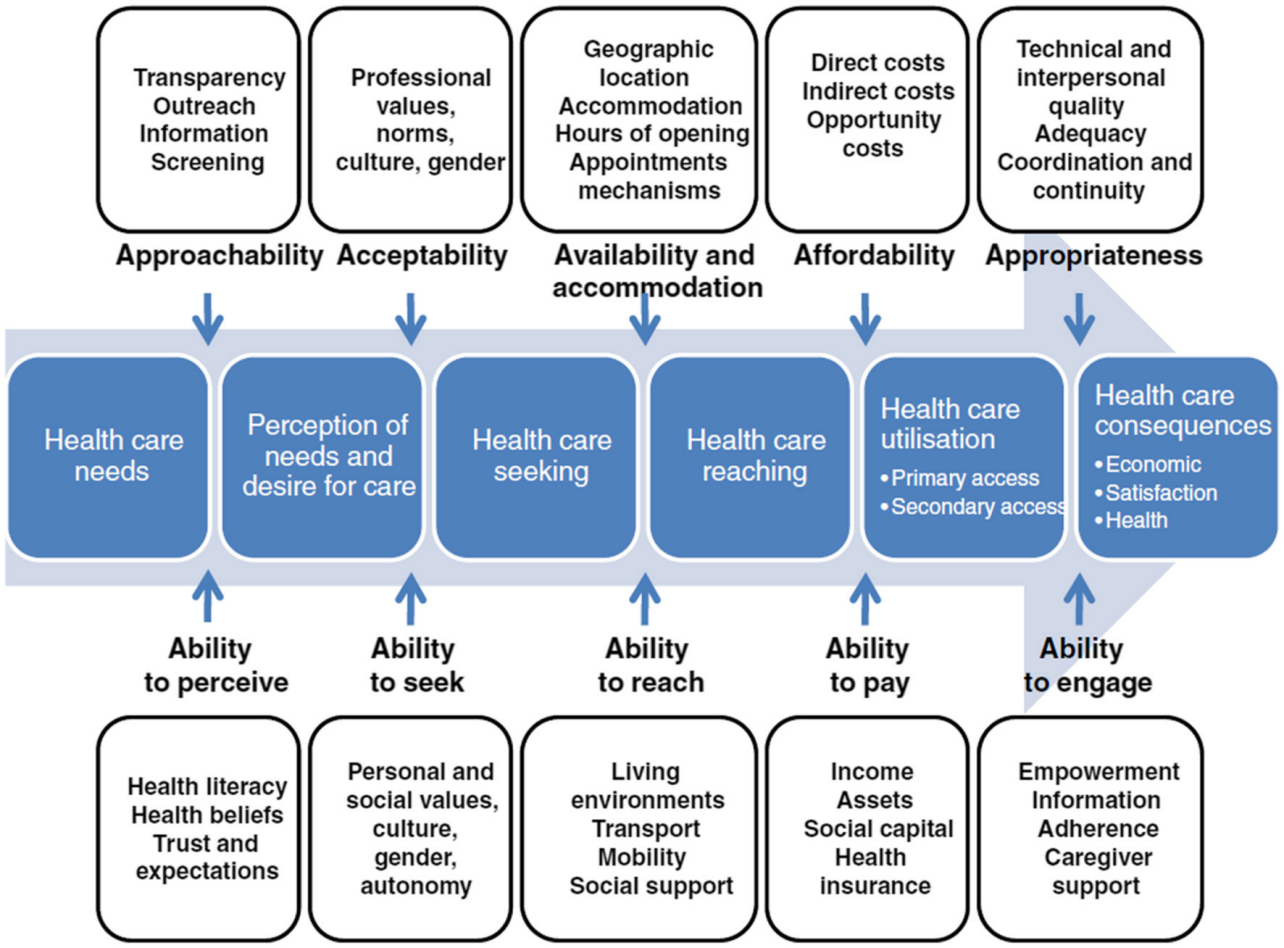
Levesque conceptual framework for healthcare access. Reproduced with permission from “A conceptual framework of access to health care” by Jean-Frederic Levesque, Mark F Harris and Grant Russell, licensed under CC BY 2.0.

“Future of Health” researchers recently identified four action areas to improve equitable access to healthcare, one of which is to foster cross-sector partnerships (71) to strengthen the capacity of community organizations to shape models of care that disrupt traditional healthcare paradigms. We heeded this call to action specifically by fostering an academic and community partnership to strengthen the capacity of the recovery community to improve access to reproductive and perinatal healthcare following Levesque's conceptual framework of access (72) (Figure 1). The dimensions of this framework encompass the 5 A's of access (approachability, acceptability, availability/accommodation, affordability, and appropriateness) as well as upstream determinants or barriers that impact each dimension. Specifically, by partnering with the recovery community, we aimed to improve the opportunities or abilities to access perinatal and reproductive health resources and healthcare that align with the 5 A's (perceive, seek, reach, pay, and engage) (72).

### Development and implementation of a champion model

This academic and recovery community partnership led to a co-created, iterative multi-level intervention model. The RCC implemented and adopted the intervention in iterative phases, with the Pregnancy Empowerment Project first (in 2022–2023) and the addition of a champion coach in the full PeRCHH model (in 2024).

The Pregnancy Empowerment Project was implemented at the organizational level of the RCC where our team trained 58 peer recovery coaches and staff primarily on the concept of how unmet reproductive health needs could impact recovery capital, how to incorporate reproductive health and autonomy into peer recovery coaching, and the rationale and basic health education of three new harm reduction items (pregnancy tests, prenatal vitamins, and emergency contraception). Each item was available from large pharmacies without a prescription in the United States, but we provided them anonymously and free in partnership with a large RCC in Kentucky. These three items served not only as key to health promotion but also as tools with the potential to expand the reach of the RCC to engage more women and participants of reproductive age to build recovery capital (47, 73). The three items were added to public-facing spaces in the RCC, events that they hosted or attended, highlighted on social media, and provided for coaches who worked with interested community partners. As reported elsewhere in greater detail, the co-creation process of development and findings from the Pregnancy Empowerment Project include increased knowledge, attitudes, and skills of the coaches and it was found to be feasible and acceptable for the role of the recovery coach and mission of the RCC (47, 73). Feedback from the focus groups then informed the next iteration we describe in this article, such as being able to make a referral to a coach with lived experience in pregnancy and SUD, more in-depth education about the three items, identifying other resources to serve women of reproductive age, and how to modify the existing recovery coaching tools to include reproductive health.

This article is the first to describe the addition of the specialized role of the peer recovery coach in championing perinatal and reproductive health and pilot data from the full model, the Perinatal, Reproductive Champion of Health and Harm reduction Program (PeRCHH). Specifically, the PeRCHH model included the continuation of the harm reduction items and RCC training from the Pregnancy Empowerment Project and a trained peer recovery coach focused specifically on engaging and reaching people who can get pregnant or are pregnant and reducing existing the barriers to health and healthcare access for this population (See Table 1 for PeRCHH training topic overview). The specialized and trained coach, referred to as the “champion” throughout the article, was first trained by the RCC as a generalist peer recovery coach and then completed an 8-h training followed by reviewing available resources and weekly check-ins by the research team.

**Table 1:** PeRCHH training.

**Topic**	**Description**
Perinatal/reproductive health promotion education	Perinatal health: preconception, conception, post-partum and interpregnancy interval. Review types of contraception. Barriers for perinatal/reproductive autonomy for women with SUD, risk periods for overdose.
Intersectional issues related to SUD and the role of RCCs	Include gender/reproductive wellbeing/justice as part of building recovery capital. Consider additional social determinants of health (violence, stigma, access) influencing recovery.
Resources to enhance learning	Reproductive wellbeing framework: 3 podcasts from the reproductive health national training center academy of perinatal harm reduction: toolkit Power to decide: resources to promote reproductive wellbeing, birth control education from Bedsider.org
How to engage	Building recovery capital, motivational interviewing, and how to apply skills to reach this population.
Linkage	Improving the 5 “abilities” to improve access, unpacking linkage to healthcare and how to advocate for participants to get the care they deserve.
Data collection	Sexual and Reproductive Health Empowerment, Brief Assessment of Recovery Capital-10, inventory of three products, field notes.
Weekly check-ins	Champion participated in a brief weekly check-in via Zoom or in-person with research team. Provided further access to knowledge or information and community partners as needed.

## Purpose

The purpose of this article was to describe multi-level outcomes from the implementation of the PeRCHH with an RCC and characterize the enhanced role of the specialized peer recovery coach (champion). We hypothesized that the PeRCHH had the potential to enhance the role of RCCs to focus on building recovery capital that was inclusive of women's reproductive and perinatal health challenges. Furthermore, the implementation of the PeRCHH would reduce barriers, increase engagement, and lead to greater access to healthcare. Specifically, we aimed to accomplish the following:

**Aim 1:** describe individual demographics, social needs, reproductive characteristics and recovery capital scores, the number of participants linked to healthcare and/or resources. Additionally, we contextualized individual data with field notes from the champion.
**Aim 2:** evaluate organizational level outcomes including pre-/post-PeRCHH engagement of women of reproductive age and reporting the uptake of the new harm reduction items as an indication of unmet needs.
**Aim 3**: characterize and contextualize the five abilities to access healthcare as they relate to the existing generalist role of the RCC coach and the addition of the champion with the PeRCHH (training and outcomes).

## Methods

### Setting/sample

The PeRCHH was implemented in partnership with Voices of Hope, a large local RCC, which serves as a community hub for recovery and harm reduction and has a broad reach of services including two mobile units that serve the state's two largest cities and trained coaches working with over 80 organizations or agencies across the state. The staff training was led by two of the authors, a perinatal nurse scientist with a clinical nurse specialty in community and public health and a trained peer research associate with lived experience, extensive experience in training peers, and national certifications in motivational interviewing. The two also led the organizational-level training sessions of the Pregnancy Empowerment Project with all RCC staff. The participant eligibility to work with the champion included being pregnancy-capable or pregnant, aged 18–45 years, who were seeking services, or would like to seek peer coaching services.

### Recruitment

Participants seeking recovery services who were pregnant or pregnancy-capable (or had other non-specified reproductive health needs such as sexually transmitted infections) were recruited by the champion, as well as referred to the champion by other RCC staff. Additionally, the research team helped the champion to network and shadow with a local perinatal clinic serving people with SUD and other community organizations serving women. The champion left behind her contact information and a referral form; the organizations agreed to let her know if she had interested participants. All intake information was collected by the champion in person, either at the RCC, or at one of the mobile sites with community partner organizations. Follow-up coaching and check-ins were both in person and over the phone at a mutually agreed-upon time frame. The majority of the interactions took place at the RCC; however, some of them also took place in the healthcare environment (neonatal unit at the hospital) or at other community organizations served by the RCC's mobile team. The champion followed the existing coaching protocols, attempted to reach the participant by phone or text three times, or to discontinue if the participant indicated the desire to terminate earlier. Participants were able to return to generalist coaching at any time beyond the three attempts and were invited to use all the RCC services regardless of enrollment status.

## Data collection

Prior to data collection, this study was approved by the Office of Research Integrity at the University of Kentucky. The champion enrolled the eligible participants and collected baseline data electronically using Qualtrics (both to guide coaching and evaluation), as well as documented elements of each peer recovery coaching session and attempts to reach a participant through RecoveryLink^TM^. The champion also took field notes to capture important elements covered during the coaching sessions such as barriers reduced, referrals made including linkage to healthcare and/or other resources (e.g., housing, transportation), as well as existing challenges or barriers. Weekly wrap-ups and successes were reported in Qualtrics. Linkage was defined as attending a healthcare appointment related to perinatal or reproductive health, such as preconception, prenatal, or post-partum appointments. The date and type of healthcare appointment was documented in the field notes. Referral to resources indicates the number of referrals to other types of resources, which were also documented in the field notes, including housing, transportation, and food insecurity. The individual-level data were only collected as part of the PeRCHH phase of the project by the champion, while the organizational level data was collected to assess change over time from the Pregnancy Empowerment Project to the end of the PeRCHH pilot.

## Measures

### Individual-level participant data

Data collected by the champion included demographics (age range, gender, race/ethnicity, sexual orientation), social needs (food insecurity, unstable housing, transportation, social support), recovery capital scores, and sexual and reproductive health characteristics. These included the number of lifetime pregnancies, miscarriages/abortions, current pregnancy status, reasons for not using birth control (including “I'd such as to get pregnant or wouldn't mind if I became pregnant”).

#### Recovery capital measure (BARC-10)

**Figure 2:**
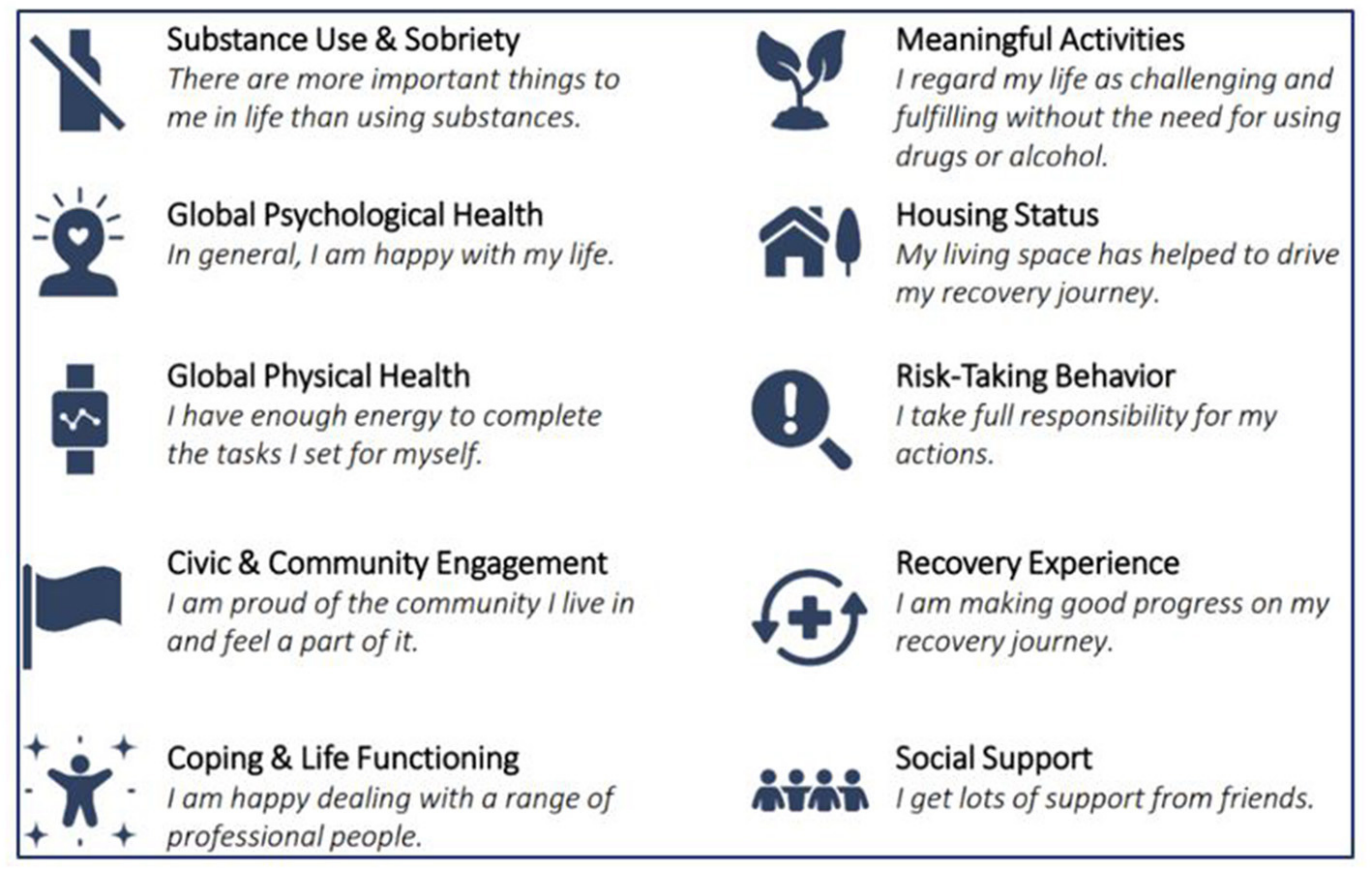
What is the BARC-10? Reproduced with permission from “Assessing Addiction Severity with the BARC-10: A Comprehensive Tool for Clinical Practice and Research”, Pennsylvania Recovery Center LLC.

The standard practice of this RCC included obtaining recovery capital scores to guide coaching every 30 days, and majority of the participants started out with weekly coaching. However, there was flexibility in the coaching schedule and was more or less frequent, based on the need and competing priorities. All programs at Voices of Hope measured recovery capital with the Brief Assessment of Recovery Capital (BARC-10). The BARC-10 is a concise, validated tool designed to assess recovery capital, which refers to the breadth and depth of internal and external resources (personal, social, and community assets) that an individual can draw upon to initiate and sustain recovery from substance use disorders (74). There are 10 domains measured by the BARC-10, with a corresponding item for each (75) (Figure 2). Each item on the BARC-10 is rated on a Likert scale ranging from 1 (strongly disagree) to 6 (strongly agree), resulting in total scores ranging from 10 to 60. Higher scores indicate greater recovery capital, suggesting more robust resources that can support the individual's recovery. The BARC-10 has been shown to be both reliable and valid in capturing the key aspects of recovery capital and is commonly used in both clinical and research settings to assess recovery-related strengths (74). A cut point of 47/60 is predictive of sustained or long-term recovery.

### Organizational level data

#### Reach

**Figure 3:**
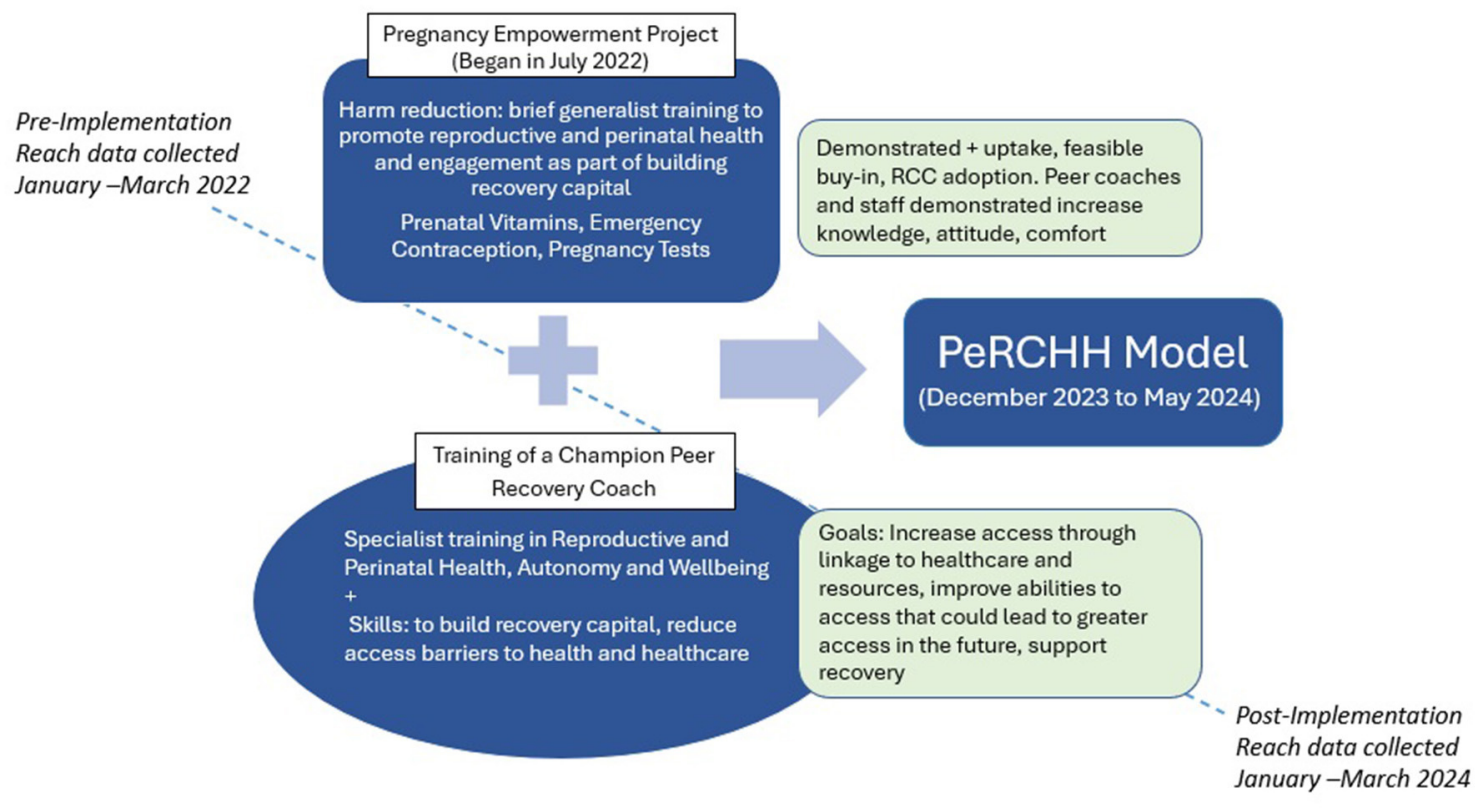
Perinatal and reproductive champion of health and harm reduction (PeRCHH) model.

Following the implementation science strategies, we defined reach as the number and proportion of people within a target population who are exposed to or engage with a program delivered by the organization (76). To assess if the RCC's reach was expanded to the target population (women of reproductive age, aged 18–45 years), we compared the number, age, and gender of all the participants served by the RCC before and after the implementation. Specifically, the quarter prior to the implementation of the Pregnancy Empowerment Project (January–March 2022) was defined as the “pre-implementation period,” and data from the last quarter of the PeRCHH implementation (January–March 2024) served as data for the “post-implementation period” (Figure 3). Additionally, we assessed the reach in terms of changes before and after the implementation of the project to the number of community partnerships or new networks to organizations that offer services primarily focused on women.

#### Uptake

The RCC tracked the monthly uptake of the three harm reduction items (pregnancy tests, emergency contraception, and prenatal vitamins) over the course of both the Pregnancy Empowerment Project and the PeRCHH intervention. In keeping with the distribution of other RCC harm reduction items, their distribution had an extremely low barrier (easy to access and anonymous), so no data were collected other than the number of items distributed. The new items were placed in the same locations as other harm reduction items (i.e., fentanyl test strips, safer smoking kits), including baskets in restrooms, meeting spaces, and mobile units; every Voices of Hope coach also could distribute the items in their off-site locations if it was appropriate and approved.

### Data analysis

Aim 1: data was downloaded and de-identified by the RCC, emailed to academic partners via a secure server, and analyzed using Microsoft Excel. The mean score and standard deviation of recovery capital were calculated, while frequencies and percentages were used for categorical data. High- and low-scored items were characterized with examples from the coaching notes.

Aim 2: reach was reported as the number of people served pre- and post-program and the proportion was indicated as a percentage. Uptake was reported as the number of products distributed or provided, and 3 months of data was averaged to account for possible seasonal differences or delays in shipments.

Aim 3: we used Levesque's conceptual framework of access to explore and contextualize the training content, existing coaching roles as they relate to building recovery capital, and champion actions taken from field notes. We identified the key components to access as they relate to the abilities of women with SUD to perceive, seek, reach, pay, or engage in healthcare or health promotion resources.

## Results

### Individual level

#### Sample characteristics

The champion enrolled 28 participants in recovery coaching from December 2023 to May 2024. All of the participants were identified as women, between the ages of 18 and 45 years; over half of the participants were younger than 35 years (54%). The majority of the participants were white (82%), non-Hispanic (93%), heterosexual (60%), and Medicaid-insured (96%). Participants reported an average of two social needs each, with transportation as the most frequently reported (61% of the participants), followed by unstable housing (46%), lack of social support, and food insecurity (29%) (see Table 2).

**Table 2:** Individual level demographic, social, recovery capital and reproductive health characteristics (*n* = 28).

**Measure**	**Item**	**Count**	**Percentage**
Age	18–24	3	10.7%
	25–35	12	42.9%
	36–45	13	46.4%
Race	More than one race	5	17.9%
	White	23	82.1%
Ethnicity	Hispanic/Latinx	2	7.1%
	Not Hispanic/Latinx	26	92.9%
Sexual Orientation	Bisexual/Pansexual	8	28.6%
	Gay/Lesbian	2	7.1%
	Prefer not to answer	1	3.6%
	Straight	17	60.7%
Top four social needs (select all that apply)	Transportation	17	60%
	Unstable housing	13	46%
	Social support	8	29%
	Food insecurity	8	29%
Measure (range)	Total	Mean per person	Percentage
Lifetime pregnancies (1–15)	131	4.7	100%
Unintended pregnancies^*^ (1–14)	106	3.9	81%
Miscarriages (0–8)	48	1.7	36.6%
Abortions (0–2)	10	0.4	7.6%
Measure	Item	Count/Range	Mean (SD) or %
Recovery capital^*^ (possible range 10–60)	BARC-10	28–60	49.9 (8.57)

(^*^1 missing).

#### Reproductive health characteristics

All participants reported having at least one pregnancy (range 1–15 pregnancies, see Table 2). Across the sample, the participants reported 131 lifetime pregnancies, 106 (81%) of which were identified as unintended pregnancies (mistimed, unwanted, or unplanned). Five were currently pregnant (18%), three had tubal ligations, and two had long-acting reversible contraception [intrauterine device (IUD) and arm implant]. These five without preconception or contraceptive needs were included in the eligibility as they identified as having a “different reproductive health issue,” which were unspecified in the notes. Eighteen participants were identified as pregnancy-capable, six of whom (33%) would like to or “wouldn't mind” getting pregnant. The remaining 12 who did not want to become pregnant were not using consistent contraception; one reported using condoms occasionally. The most frequently reported reason to not use consistent birth control (select all that apply) was “I don't have sex very often” by nine participants, followed by “I don't have time or interest in seeking birth control as I have a lot of other needs” by four participants, and “I don't like the side effects” by three participants. There were no follow-up questions to distinguish if there was a potential for pregnancy related to the kind of sex they were having.

#### PeRCHH champion coaching outcomes

The 28 participants received 147 coaching sessions, with an average of five coaching sessions per participant (range 0–17). The champion was able to refer participants to 58 different community resources to address a wide range of needs, such as housing, employment, clothing, emergency childcare, and food, and provided bus passes or gas cards when the RCC had them available. On average, she made two referrals for social needs per participant. Twenty participants reported unmet reproductive or perinatal health needs, and the champion was able to successfully reduce barriers and link five participants to healthcare (25%), confirming an appointment date and attendance with the participant.

#### Recovery capital and coaching

The baseline recovery capital scores were relatively high (mean score = 49.9), with 18 participants scoring 47 or above, which is considered predictive of long-term recovery (74). Only five (18%) completed a follow-up recovery capital score at 1 month (mean score=54.9) and four (14%) completed a 3-month recovery capital score (mean score = 53.0). We were unable to meaningfully examine the differences between baseline and follow-up; however, they seemed to improve from baseline for those who completed the 3 months of coaching.

The two highest scored items at baseline were in the domains of Risk-Taking Behavior (“I take full responsibility for my actions”) and Recovery Experience (“I am making good progress on my recovery journey”). The champion coaching notes revealed alignment with these items in that women would celebrate their victories and be proud of the milestones they were achieving. They described sobriety and pregnancy milestones, reduction in drug use, promotions at fast food jobs, and celebrating children's birthdays when they couldn't be present in past years due to their drug use. The champion would leverage these strengths and values in her coaching when there were opportunities related to planning their ideal family size or making healthy decisions for the future they deserve or want for their children.

The lowest scored item was in the Global Physical Health domain (“I have enough energy to complete the tasks I set for myself”). The next lowest item was in the Civic and Community Engagement domain (“I am proud of the community I live in and feel a part of it”). The champion coaching notes revealed coping challenges that impacted their energy levels and recovery capital including partner violence, legal and child protective services issues, work demands, and transportation issues. Many participants who were in the early recovery stage struggled with their temporary housing situations, often referring to the strict rules or gossiping at their “sober living” houses or shelters or not being able to bring their pets or things that provided them comfort. These could certainly have an impact on the way they feel about the community they are currently a part of. The champion was able to provide social support, get them connected with RCC resources such as the yoga class, as well as an organization that provides emergency childcare so they could attend job interviews, go to court, or go to work.

While the number of currently pregnant women enrolled was small (five out of 28), their recovery needs tended to be different than those not pregnant. They averaged lower recovery capital scores (40.4) and their lowest scored item was related to their housing status (“My living space has helped drive my recovery journey”). Pregnant participants also averaged a higher number of champion coaching sessions (9.5). Champion coaching notes revealed that there were challenges with housing; many participants were trying to stay away from their former social networks and places where they could stay prior to the pregnancy. They reported being afraid they would use drugs again if they went back to their communities. The champion was asked to visit two participants in the hospital after childbirth because they didn't have any visitors, and they wanted an advocate who had similar experiences. Another pregnant participant was asked to leave her ‘sober living house' because she was in her 7th month of pregnancy and their insurance didn't cover this level of care because there was no nurse on-site. The champion was unable to find her immediate temporary housing, and this participant was living in her car with her partner and went into pre-term labor. She called the champion for support and to visit her at the hospital in the intensive care unit (ICU) to where her newborn was transferred.

### Organizational level

#### Reach

Overall, participation in RCC services increased from before to after the study by 54%, and the RCC grew substantially unrelated to this project. Our target population (women 18–45 years) had the greatest proportional increase (66%) in the participation in RCC services (Table 3). Additionally, the RCC reported adding nine additional partnerships/referral sites with organizations that primarily serve women in the same time period. These included organizations that serve victims of partner violence, pregnant and parenting people (providing case management, emergency childcare, and resources for newborns), and new healthcare partnerships with family planning, midwife, and OB/GYN clinics.

**Table 3:** Organizational level outcomes: reach by gender pre/post.

**Item**	**January–March 2022 (pre)**	**January–March 2024 (post)**	***n* (%)**
Men (all ages)	186	273	87 (47%)
Women (all ages)	155	219	64 (41%)
Women (18–45)	113	188	75 (66%)
Non-binary, transgender, or unknown	4	25	21

#### Uptake

The uptake of the additional harm reduction items was brisk throughout the entire project, indicating an unmet need in the community. Starting with the Pregnancy Empowerment Project phase (July 2022), the three items were initially limited to the RCC and mobile units in two counties and during the period of the PeRCHH intervention, they were able to expand the availability of these items to seven additional counties where they had coaches. The average monthly uptake in the first quarter of the Pregnancy Empowerment project included 106 pregnancy tests, 104 emergency contraception medication boxes (one dose per box), and 25 bottles of prenatal vitamins (90 per bottle). In the last quarter of PeRCHH, the average monthly uptake had more than doubled, with 404 pregnancy tests, 230 emergency contraceptions, and 105 prenatal vitamins distributed/provided.

Reporting the organizational level data about the reach and the brisk uptake of new harm reduction products to serve an unmet reproductive health need does not tell the human story. We captured many of these stories in our field notes. For example, from our field notes, an RCC staff member and champion shared:

“A young woman came to the RCC and hesitantly asked for a pregnancy test. She was directed to the basket in the restroom. She said she did not want to enroll in coaching or any other services. She came back the next day with her mother, because she was pregnant and wanted a referral for [SUD] treatment, a prenatal visit and housing for victims of intimate partner violence. A coach remembered her from the day before and was able to meet with her and help her with each of these requests and supplied her with a 90-day supply of prenatal vitamins (and further explained the importance). Imagine how long some people wait to do all of those things if you have to buy the pregnancy test. These small moments of recovery support occur every day at RCCs. They are a safe place to identify and ask for what you need.”

### 5 A's findings related to access

Informed by our earlier qualitative work (46, 77) and Substance Abuse and Mental Health Services Administration (SAMHSA) Bringing Recovery Supports to Scale (78), we identified relevant roles of coaches and RCCs where their work already promotes the five abilities (perceive, seek, reach, pay, engage) as they relate to building recovery capital and recovery support. Then we aligned these five abilities with the additional components of the PeRCHH training to enhance the role of the peer recovery coach to bring these forms of support specifically to pregnant and pregnancy-capable people (Table 4). We further contextualized this activity with more explicit examples and stories of actions taken with the expansion of the role of the RCC and champion coaching as a part of the PeRCHH implementation as they relate to recovery capital and recovery support.

**Table 4:** Contextualizing “abilities” as they relate to building recovery capital and the PeRCHH.

**5A's abilities**	**Relevant recovery coaching roles to build recovery capital**	**PeRCHH model training topics**	**Examples of enhanced PeRCHH roles**
Perceive	Assess beliefs, build trust, leverage strengths	Health Promotion Education: sexual, reproductive, and perinatal health Assess risk perception related to becoming pregnant	Participants shared stories related to pregnancy, desire to become pregnant, or not to become pregnant
Seek	Assess values, family/legal challenges related to child protective services Build their sense of control/agency over their lives	Desires and values surrounding pregnancy Partner control Provide new self-care and harm reduction items to improve agency	Included reproductive and/or perinatal health in recovery goal setting Referrals to local organizations for victims of partner violence Promoted uptake of new harm reduction/ self-care items
Reach	Reduce barriers and competing priorities: housing, food, safety, transportation Identify social support, attend groups, pro-social behaviors	Seek partnerships and services to promote perinatal and reproductive health and autonomy	Referrals or linkage to: WIC, pregnancy crisis support, housing for people who have been sexually exploited, for single moms/full-time students, parenting classes and support Maternal SUD case management
Pay	Assess insurance status: apply for Medicaid/find free or sliding scale healthcare Reduce barriers to employment: ID card, enroll or move schools for children	Reviewed birth control methods, Title X clinic search tools, and expanded Medicaid coverage for post-partum care.	Provided maternity clothing for employment Connected to employment training: for some to make progress toward obtaining the resources and structure to regain child custody
Engage	*Use motivational interviewing skills to:* Resolve ambivalence, strengthen motivation and commitment to change Reduce barriers such as fear and stigma Help participant call for appointments, find child-care	*Use motivational interviewing skills to:* build skills to enable empowerment tied in reproductive well-being Promote advocacy and trauma informed care at their appointment Role played conversations for appointments/advocacy	Linkage to primary, reproductive, and perinatal healthcare Attended prenatal, post-partum and hospital visits with participants when possible.

## Discussion

PeRCHH offered a gender-inclusive approach to build women's recovery capital through linkage to social resources and access to reproductive and perinatal healthcare, as well as potentially improving organizational reach to serve more women of reproductive age with recovery services. One quarter of the participants were linked to perinatal and reproductive healthcare, which confers a cascade of health benefits and leads to more equitable outcomes for people with SUD. These outcomes are critical to public health, including the prevention of maternal overdose and mortality, unintended pregnancy, birth defects, pre-term delivery, and infant mortality (36–38, 79).

There were several lessons learned from the implementation of this model. First, in line with the existing literature (80, 81), transportation was the most commonly reported barrier. Moreover, this barrier may particularly impact access to reproductive health services because women are more likely to report delaying healthcare than men due to issues with transportation (82). Women may have greater challenges with transportation due to childcare responsibilities and real and perceived issues around personal safety (83), including financial and emotional abuse by intimate partners (84). Furthermore, other vulnerable groups, such as those with poorer health status are more impacted by transportation challenges. Therefore, there is a need to consider creative transportation solutions, such as providing bus passes as well as incorporating peer-driver provided rides to healthcare services (85).

Additionally, the PeRCHH intervention included the in-depth training of one champion coach. This provided benefits, such as developing more in-depth knowledge of perinatal and reproductive wellbeing, developing relationships with clinical and community partners that help to fast-track some appointments, and having one coach to go to for questions and referrals. However, having just one champion also has limitations. In her absence, other RCC coaches did not have comparable training, which made it difficult to fill in and share the tasks. Future studies could test the impact of training two champion coaches.

Finally, the PeRCHH was co-led by people with lived experience, and the intervention was informed by qualitative interviews, focus groups, and the Survivors Union of the Bluegrass, a University of Kentucky community advisory board comprising people who use drugs (47, 73, 86). Within our model, people with lived experience guided the intervention development and delivery, and the academic partners provided scientific support, determined the measurement outcomes to evaluate, developed tools to test the intervention, sought funding when asked, and helped to develop sustained community and healthcare partnerships. The integration of people with lived experience across all the elements of this study is in line with best practices for the successful implementation of community interventions (87).

## Limitations

A limitation of this study is that the sample is not reflective of minoritized populations such as black women, who have among the highest maternal mortality in the United States, and whose process of recovery may be unique with intersectional challenges we were not able to include (88, 89). While we were able to recruit over one-third of participants with minoritized sexual orientations, all the participants identified themselves as women, and we were unable to recruit pregnant or pregnancy-capable participants with gender-expensive identities. Tailored and inclusive marketing materials or targeted outreach strategies could be improved in future iterations. Furthermore, while PeRCHH is evidence-informed and promising, we were not able to examine the efficacy of the pilot model as we did not enroll enough participants in the PeRCHH champion coaching longitudinally to assess improvements in recovery capital over time and did not assess any participant health benefits.

## Conclusion

PeRCHH provided promising strategies to build recovery capital by enhancing the role of RCCs in improving access of people with SUD to reproductive and perinatal health and healthcare. Improving access to reproductive and perinatal healthcare for women with SUD with our community-embedded model has the potential to set the foundation for healthy pregnancies, improve the health of women and families, reduce maternal mortality, and promote long-term recovery.

## Data Availability

The raw data supporting the conclusions of this article will be made available by the authors, without undue reservation.
